# Effectiveness of a supportive care program via a smartphone application on the quality of life and care burden among family caregivers of patients with major depressive disorder: a randomized controlled trial

**DOI:** 10.1186/s12889-023-17594-4

**Published:** 2024-01-02

**Authors:** Somaye Minaei-Moghadam, Zahra Sadat Manzari, Saeed Vaghee, Seyedmohammad Mirhosseini

**Affiliations:** 1 Nursing and Midwifery Care Research Center, Mashhad University of Medical Sciences, Mashhad, Iran; 2 Department of Nursing, School of Nursing and Midwifery, Shahroud University of Medical Sciences, Shahroud, Iran

**Keywords:** Family caregivers, Care burden, Quality of life, Smartphone application, Major depressive disorder

## Abstract

**Background:**

The majority of patients with major depressive disorder require care that has generally affected caregivers’ lives. Providing care could cause negative experiences as a care burden and deteriorate quality of life. However, there is a lack of evidence about caregiver training-based informatics and its impact on the caregiver’s life.

**Methods:**

This experimental study was carried out in Mashhad, Iran. A total of 60 primary family caregivers of patients with major depressive disorder were included in the study between February and July 2021. The quadruple block randomization method was used to allocate the participants into control and intervention groups. In the intervention group, family caregivers used the application with weekly phone calls for one month. The app contains the most important points of patient care and has the possibility of communicating with the nurse. The Novak and Guest Care Burden Inventory and the short form of the World Health Organization Quality of Life Questionnaire were completed before and after the intervention. Data analysis was performed using chi-squared tests, independent sample t tests, and analysis of covariance.

**Results:**

At baseline, the mean scores of care burden and quality of life were homogeneous between the two groups. After the intervention, the mean scores of care burden and quality of life were significantly reduced and improved in the intervention group compared with the control group (*p* < 0.001).

**Conclusions:**

Using the application with the ability to communicate with the caregiver, along with educational support, helps to strengthen the relationship between the family caregiver and the nurse. Despite the effectiveness of the present intervention, before including this form of implementation of support in care programs, it is necessary to evaluate its other positive aspects in future studies.

**Trial registration:**

Iranian Registry of Clinical Trials (IRCT), IRCT20210202050222N1. Registered on 05/02/2022.

## Background

Depression is considered an individual and social crisis due to its high prevalence and debilitating nature [1]. Based on previous studies, major depressive disorder (MDD) has a point prevalence of 12.9% and a lifetime prevalence of 10.8% worldwide. Also, in Iran, its prevalence was estimated equal to 4.8% and 2.3% in women and men respectively [2, 3]. Worldwide, approximately one-third of people with depression receive treatment. Three out of five patients are treated. This is even though usually, the treated patients have not received at least enough treatment [4]. Depression treatment rates are very low in low- and middle-income countries [5]. Depression negatively affects various aspects of life [6, 7]. A person suffering from depression is involved with symptoms such as changes in appetite and weight, changes in sleep and activity, problems in communication, recurrent thoughts of suicide, and its extensive impact on various aspects of life [8]. Individuals with MDD often need the support and involvement of family members to overcome the challenges associated with the disorder. Having a strong support system can assist individuals in managing their symptoms and obtaining the necessary treatment [9].

On the other hand, providing care for these patients has affected their caregivers’ lives. Lower quality of life levels in caregivers seem to be associated with a decrease in patient acceptance [10] and affect rehabilitation, the patient’s quality of life, and disease complications [11]. Quality of life involves conditions such as satisfaction, happiness, feeling of satisfaction, and pride in one’s life, and high quality of life is usually defined as satisfaction and the ability to overcome problems [12, 13]. caregivers of individuals with MDD have significantly lower Quality of life (QoL) compared to the general population. The emotional, social, and physical well-being of caregivers appears to be particularly affected [14].

It brings the caregiver’s quality of life in view as an important outcome of caring considering that quality of life has been directly affected by the caregiver’s care burden [15]. Care burden follows the caregiving experience that is a physical, psychological, social, or economic reaction during caregiving, and it appears to be a negative experience caused by providing care to patients [16]. The caregiver’s lack of awareness of the disease and proper care for the patient can make caring time-consuming and even costly [17]. At the same time, lack of awareness is one of the factors inhibiting caring, increasing the care burden, and decreasing QoL [18, 19]. On the other hand, caregiver training and support can improve the symptoms and complications of the disease in the patient [20, 21]. Based on the literature review, significant levels of caregiving burden and impaired quality of life have been reported in Iranian family caregivers of patients with MDD [22–24]. The majority of these caregivers are not familiar with the patient’s disease, symptoms, or care-providing requirements [25]. This issue for the caregivers of patients with mental diseases affects the way they perceive their patients and makes it complicated [26]. Empowering caregivers to recognize early symptoms, monitor behavioral changes, and provide care to support patients with psychosis has been observed [27]. Additionally, the caregivers of suicidal patients had the desire to participate and train more in the professional care of their patients. A demand-based intervention tailored to the family in addressing the needs of the caregivers regarding the disease, along with developing effective coping strategies for the caregivers of the patients with mental disease, has been recommended [28]. Teaching basic care skills along with psychological skills can increase people’s care capacity [29], and supporting families, as the most important caregivers of the patient, seems beneficial for improving the care gap [30].

The smartphone application provides the transfer of concepts and content extensively and attractively by using media capacities such as using video, photo, and sound along with written content that leads to a better understanding. On the other hand, this continuous educational content is available to the caregiver and saves time and money [31].

Based on the literature review, the use of smartphone applications can provide the necessary support to patients and their caregivers in nursing care and public health to provide support and improve well-being and quality of life [32, 33]. In Otero’s (2020) study, conducting an intervention with a brief psychological approach through a smartphone application was effective in reducing caregivers’ depression symptoms [34]. Other studies in this regard have also benefited from this type of support intervention to improve the quality of life and reduce the burden of care for other diseases [35, 36]. Nevertheless, most of the evidence related to interventions based on mobile phone applications has focused on chronic physical diseases, and less has addressed the caregivers of patients with mental disease. Considering the restrictions of the coronavirus, which has led to a decrease in communication between the nurse and the family, it seems that it is possible to respond to the needs of the caregivers with this method. In the software banks of smartphones, no application was found with care content for the caregivers of depressed patients. Other applications were checked based on the content. None of them were found to contain evidence-based content for helping the caregiver. Therefore, the present study aimed to investigate the effectiveness of a supportive care program via a smartphone application on the quality of life and care burden among family caregivers of patients with major depressive disorder.

## Methods

### Study design, settings, and participants

The participants of the present experimental study consisted of the main family caregivers of patients with MDD who were referred to Patient Education Clinic of Ibne-Sina Hospital (one of the largest psychiatric referral centers in Iran) from February to July 2021. Caregivers over the age of 18 years who owned a smartphone and were able to use smartphone applications based on their self-reports and the researcher’s review at the time of application installation were included in this study and were the main caregivers of the patient (which was determined by at least having two criteria: following the patient’s treatment process, living with the patient at same place, and spending at least an average of four hours a day and 24 h a week with the patient). If the mobile phone was out of reach for any reason or if caregivers participated in any educational program interventions during the past months, they were excluded from the study. Patient inclusion criteria included age between 18 and 60 years, diagnosis of depression by a psychiatrist, and absence of acute physical diseases. When the patient was hospitalized in a psychiatric or general hospital, his or her caregiver was excluded from the study.

**Fig. 1:**
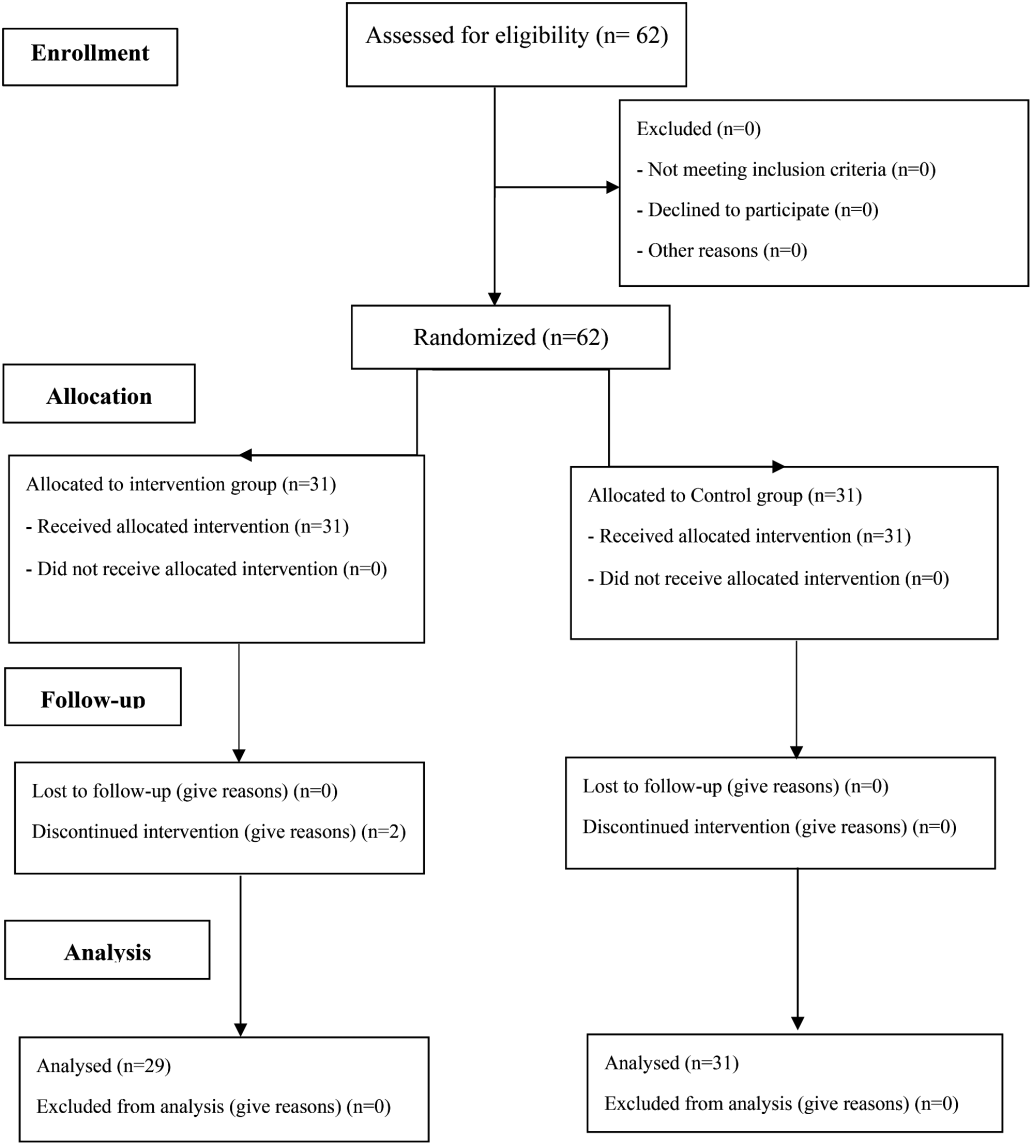
The flow diagram of study

A total of 62 caregivers were recruited in the study using the convenience sampling method. Two caregivers were excluded from the study due to the refusal to continue participating (Fig. 1). The statistical consultant generated the random allocation sequence. First author enrolled the participants and the trained nurse assigned them to intervention. In the present study, random allocation was done using quadruple block method. Random allocation sequence was obtained using SPSS software. Also, the data were collected by the research assistant who did not know the random allocation in participants.

### Tools

The data were collected using self-report tools. The variables examined in this study included the personal information of the caregivers and patients, care burden, and QoL. The sociodemographic questionnaire included the age of the patient and the caregiver, sex of the patient and the caregiver, level of education, marital status, employment status, time spent with patients, caregiver’s relationship with the patient, presence of psychotic symptoms, family history of mental diseases, time since diagnosis, and frequency of disease recurrence in the last year.

The Novak and Guest’s Caregiver Burden Inventory (CBI) was used to examine the caregiver care burden, which has been used in the context of chronic diseases [37]. This questionnaire contains 24 items and five dimensions, which include time dependence care burden (5 items), developmental care burden (5 items), physical care burden (4 items), social care burden (5 items), and emotional care burden (5 items). Scoring is based on a five-point Likert scale, ranging from strongly disagree (1) to strongly agree (5). The final score ranges between 24 and 120, with higher scores indicating a higher care burden. The original version of this instrument reported Cronbach’s alpha coefficients between 0.73 and 0.87 [16]. The reliability of this tool for the caregivers of Alzheimer’s patients was estimated at 0.93, based on the internal consistency coefficient (Cronbach’s alpha) [38]. In our study, the Cronbach’s alpha coefficient was 0.88.

Additionally, the World Health Organization Quality of Life Questionnaire-Short Form (WHOQOL-BREF) is a 26-item questionnaire that assesses a person’s general QoL in four subscales. The first two questions do not belong to any of the subscales and examine overall QoL/health. Subscales include physical health, mental health, social relationships, and environmental health. The total score obtained ranged from 26 to 130, with higher scores indicating a higher QoL. To determine reliability, internal consistency based on Cronbach’s alpha equal to 0.89 was calculated [39]. The previous study by Usefy et al. (2010) reported Cronbach’s alpha values between 0.76 and 0.82 for four domains of the WHOQOL-BRE [40]. In our study, the Cronbach’s alpha coefficient was 0.79.

### Intervention

The first stage was the preparation of the care content. The classification was based on the nursing diagnosis of MDD patients. For this purpose, the book Psychiatric-Mental Health Nursing and the International Care guideline were reviewed (http://www.apna.org, http://www.nanda.org, http://www.ispn-psych.org) [41, 42]. An expert panel of 12 faculty members consisting of five psychiatric nurses, three psychiatrists, and four professors of nursing suggested their viewpoints about the application’s contents and care plans. The researchers revised them based on their comments.

The content of care included the following areas: nutrition, medications and psychotherapies, sleep hygiene, regulation of activities, definition of depression and its related disorders, symptoms of major depressive disorder, and suicidal thoughts and related cares, diagnostic measures, communication strategies to improve the patient-caregiver relationship, and electroconvulsive therapy and related care. It was possible for each caregiver to access all sessions at the same time. The second stage was the preparation of the smartphone application. With the cooperation of an information technology specialist, the application environment was designed based on the final content. The application was designed in the Android Studio Environment for Android smartphones version 5 (and above) using several programming languages ​​(HTML, CSS, JavaScript, jQuery, Ajax, Java, PHP, and MySQL). The designed application was hybrid, and its overall structure contained six separate menus, in addition to a management panel in the form of a support website for the application. The application had various options, including multimedia care content (audio, videos, and photos), medication time reminders, and communication with the researcher in a chat section so that the caregiver could communicate with the nurse at a certain time (4–6 pm) if they had any questions.

The initial application was evaluated by the informatics specialist using white-box testing or structural testing to examine the performance of each section. Other possible interactive errors were then examined in the application using black-box testing or behavioral testing. After structural approval, the application was presented to two professors of the nursing school, a software engineer, and a psychiatric nurse for further review to examine the technological aspects and transfer of information. Next, it was presented to five caregivers to evaluate its functionality. Moreover, the Mobile App Rating Scale (MARS) was employed to evaluate the application by the participants [43]. This scale included 23 questions in five areas, including engagement, functionality, aesthetics, information, and mental quality. The items were scored based on a five-point Likert scale. A total score of MARS was 4.04 ± 0.73.

Finally, the third stage was the implementation of the intervention over four weeks. In the intervention group, the application was installed for the family caregivers in a 15- to 30-minute session individually, and they were provided with the necessary information. After installing the application and explaining its use, the caregivers were allowed to ask any questions about different sections of the application. Additionally, during the study, they could ask any questions in the chat section. In addition, they were contacted weekly via phone calls for one month. To prevent the spread of information in the intervention group, a password was assigned to the application so that it would not be opened by anyone other than the participants. In the control group, all routine intervention and actions of Patients Education Clinic were performed by clinic manager who was a nurse. In other words, due to restrictions related to the COVID-19 pandemic during the study, the patient’s caregivers in this hospital received training by a nurse in a 10- to 15-minute face-to-face session using pamphlets upon discharge and supported by weekly phone calls; training focused on the time of medication use and important side effects.

### Sample size

The sample size was estimated to be 60 based on previous research [44, 45] by considering the mean and standard deviation of the main variables (care burden and quality of life). A confidence level of 95% and a power of 0.8 were considered. The sample size was determined using the standard formula.

$$ \frac{{({\text{Z}}_{1-{\alpha }/2}+ {\text{Z}}_{1-{\beta }})}^{2}\times\left[\left({{\text{S}}_{1}}^{2 }+ {{\text{S}}_{2}}^{2 }\right)\right]}{{({X}_{1}- {X}_{2})}^{2}}$$

### Blinding

In the present study, based on the nature of the intervention, participants were not blinded. Additionally, the statistical consultant and data collector were blinded regarding the random allocation.

### Data analysis

The normal distribution of the data was assessed by Kolmogorov-Smirnoff test. Data analysis was performed according to parametric analysis based on the normal distribution. Descriptive statistics (frequency, percentage, mean, and standard deviation) were measured to describe and categorize the data. Additionally, inferential statistics were calculated to test the study hypothesis. To examine the homogeneity of background variables in the two groups (e.g., demographic and main variables), the chi-squared test and independent sample t test were performed. Additionally, an analysis of covariance (ANCOVA) was performed to eliminate the effect of the pre-intervention mean score and group variable. In all tests, a significance level of 0.05 was considered. Data were statistically analyzed by SPSS 25.

## Result

The average age of the caregivers in the intervention and control groups was 38.4 ± 9.1 and 39.9 ± 8.9, respectively (p = 0.532). The majority of caregivers were female (N = 44). Most of the participants were married (including 42 participants). Additionally, the caregivers in both groups were homogeneous in terms of their relationship with the patient (p = 0.890) and the duration of daily care (p = 0.982), as the majority of the caregivers (41.7%) had a spousal relationship with their patient. Additionally, they took direct care of their patients for 7.5 ± 12.9 h per day. Additional results are presented in Table 1.

**Table 1:** Demographic characteristics of participants

Variables		Intervention	Control	*p*
		n (%)	n (%)	
Gender	Male	10 (34.5)	6 (19.4)	0.185^*^
	Female	19 (65.5)	25 (80.6)	
Marital status	Single	10 (34.5)	8 (25.8)	0.464^*^
	Married	19 (65.5)	23 (74.2)	
Level of education	Illiterate or elementary level	0 (0.0)	1 (3.2)	0.831^*^
	Secondary school	12 (41.4)	10 (32.3)	
	High school	11 (37.9)	14 (45.2)	
	Academic degree	6 (20.7)	6 (19.3)	
Employment status	Housewife	11 (37.9)	15 (48.4)	0.609^*^
	Unemployed	0 (0.0)	1 (3.2)	
	Self-employed	7 (24.1)	6 (19.4)	
	Employee	11 (37.9)	9 (29.0)	
Relation with the patient	Mother	4 (13.8)	7 (22.6)	0.156^*^
	Father	1 (3.4)	1 (3.2)	
	Child	5 (17.2)	5 (16.1)	
	Wife/husband	12 (41.4)	13 (42.0)	
	Sibling	7 (24.2)	5 (16.1)	
		Mean ± SD	Mean ± SD	
Age (year)		38.4 ± 9.1	39.9 ± 8.9	0.532^**^
Duration of daily care (hour)		12.8 ± 7.8	12.9 ± 7.4	0.982^**^

^*^ Chi-squared test

^**^ Independent t-test

n: frequency; %: percent; SD: standard deviation

Furthermore, the average age of patients in both intervention and control groups was 39.1 ± 12.1 and 38.8 ± 13.0 (p = 0.911). Between the patients of the two groups, in terms of the presence of psychotic symptoms (p = 0.152), family history of mental disorders (p = 0.275), duration of the disease (p = 0.588), and frequency of disease recurrence in the last year (p = 0.165), no significant difference was observed. The results of the present study showed that the patients in intervention and control groups were homogeneous in terms of demographic variables such as gender, marital status, employment, and educational status (p > 0.05). (Table 2)

**Table 2:** Demographic information of patients with major depressive disorder

Variable		Intervention	Control	*p*
		n (%)	n (%)	
Gender	Male	14 (48.3)	15 (48.4)	0.993^*^
	Female	15 (51.7)	16 (51.6)	
Marital status	Single	13 (44.8)	8 (25.8)	0.123^*^
	Married	16 (55.2)	23 (74.2)	
Level of education	Illiterate or elementary level	8 (27.8)	17 (54.9)	0.123^*^
	Secondary school	12 (41.4)	8 (25.8)	
	High school	4 (13.8)	1 (3.2)	
	Academic degree	5 (17.2)	5 (16.1)	
Employment status	Housewife	15 (51.7)	19 (61.3)	0.616^*^
	Unemployed	13 (44.8)	10 (32.3)	
	Self-employed	0 (0.0)	1 (3.2)	
	Employee	1 (3.4)	1 (3.2)	
Psychotic symptoms	Yes	4 (13.8)	9 (29.0)	0.152^**^
	No	25 (86.2)	22 (71.0)	
Family history of mental disorders	Yes	19 (65.5)	16 (51.6)	0.275^*^
	No	10 (34.5)	15 (48.4)	
		Mean ± SD	Mean ± SD	
Age (year)		39.1 ± 12.1	38.8 ± 13.0	0.911^***^
Duration of the disease (month)		17.8 ± 11.7	19.3 ± 10.0	0.588^***^
Number of disease recurrence in the last year		2.6 ± 1.2	2.1 ± 1.2	0.165^***^

^*^ Chi-squared test

^**^ Fisher’s exact test

^***^ Independent t-test

n: frequency; %: percent; SD: standard deviation

According to the results of the independent t test, no significant difference was observed between the mean scores of care burden before the intervention between the control and intervention groups (p = 0.778). After the intervention, a significant difference was observed between the groups, and the mean scores of care burden in the intervention group were significantly lower than those in the control group (p < 0.001). Additionally, a significant difference was observed in the mean differences of care burden between the two groups, so that in the intervention group, the care burden score decreased more after the intervention compared to the control group (8.5 ± 12.6 versus 6.1 ± 2.1). (Table 3)

**Table 3:** Mean scores of care burden and its subscales before and after the intervention in both groups

Variables		Groups		*p* ^*^
		Intervention (n = 29)	Control (n = 31)	
		Mean ± SD	Mean ± SD	
Time-dependent care burden	Pre-intervention	18.1 ± 3.0	19.1 ± 2.7	0.169
	Post-intervention	14.7 ± 1.9	20.0 ± 2.2	< 0.001
	Mean differences	−3.4 ± 2.4	0.9 ± 2.6	< 0.001
Developmental care burden	Pre-intervention	18.5 ± 3.1	18.3 ± 2.4	0.813
	Post-intervention	16.6 ± 2.2	18.4 ± 1.9	< 0.001
	Mean differences	−1.9 ± 2.5	−0.1 ± 3.1	0.008
Physical care burden	Pre-intervention	15.1 ± 2.7	15.5 ± 2.9	0.565
	Post-intervention	12.6 ± 2.5	15.8 ± 2.3	< 0.001
	Mean differences	−2.4 ± 2.5	0.3 ± 1.8	< 0.001
Social care burden	Pre-intervention	18.4 ± 3.8	18.3 ± 3.2	0.891
	Post-intervention	16.6 ± 3.7	18.2 ± 2.7	0.051
	Mean differences	−1.8 ± 2.4	−0.1 ± 1.5	< 0.001
Emotional care burden	Pre-intervention	18.6 ± 2.7	16.5 ± 3.6	0.016
	Post-intervention	15.6 ± 2.4	17.4 ± 3.6	0.034
	Mean differences	−3.0 ± 7.8	0.8 ± 1.1	< 0.001
Total	Pre-intervention	88.7 ± 13.7	87.8 ± 11.2	0.778
	Post-intervention	76.1 ± 8.8	89.8 ± 7.3	< 0.001
	Mean differences	−12.6 ± 8.5	−2.1 ± 6.1	< 0.001

^*^ Independent t-test

n: frequency; P: P-value; SD: standard deviation

The results of the present study showed that the mean scores of the QoL before the intervention were not significantly different between the groups (p = 0.320). In addition, after the intervention, the mean QoL scores in the intervention group were significantly higher than those in the control group (p < 0.001). On the other hand, a significant difference was observed between the mean scores of the QoL between the two groups (p < 0.001). As in the intervention group, QoL scores increased, and in the control group, they decreased (4.8 ± 2.9 versus 5.3 ± 12.3). (Table 4)

**Table 4:** Mean scores of quality of life and its subscales before and after the intervention in participants

Variables		Groups		*p* ^*^
		Intervention (n = 29)	Control (n = 31)	
		Mean ± SD	Mean ± SD	
Physical health	Pre-intervention	18.9 ± 3.0	19.4 ± 4.9	0.639
	Post-intervention	21.5 ± 3.31	18.1 ± 4.1	< 0.001
	Mean differences	2.6 ± 2.1	−1.3 ± 2.2	< 0.001
Psychological health	Pre-intervention	17.1 ± 2.4	17.5 ± 3.3	0.611
	Post-intervention	18.1 ± 3.3	17.1 ± 2.6	0.201
	Mean differences	1.0 ± 2.0	−0.4 ± 2.4	0.019
Social relationship	Pre-intervention	7.8 ± 2.0	8.4 ± 2.1	0.321
	Post-intervention	11.0 ± 2.6	8.1 ± 2.1	< 0.001
	Mean differences	3.2 ± 2.7	−0.3 ± 0.8	< 0.001
Environmental issues	Pre-intervention	21.0 ± 3.2	22.6 ± 5.0	0.133
	Post-intervention	25.5 ± 2.7	21.7 ± 4.6	0.007
	Mean differences	4.5 ± 3.5	−0.9 ± 1.4	< 0.001
Quality of life	Pre-intervention	70.9 ± 8.3	73.8 ± 13.6	0.320
	Post-intervention	83.2 ± 7.1	70.9 ± 11.5	< 0.001
	Mean differences	12.3 ± 5.3	−2.9 ± 4.8	< 0.001

^*^ Independent t-test

n: frequency; P: P-value; SD: standard deviation

In addition, based on the results presented in Table 4, the factors affecting the QoL and care burden mean scores after the intervention were evaluated based on analysis of covariance (ANCOVA). The results showed that the mean QoL score before the intervention and the group variables affected the mean QoL score after the intervention. The patients in the intervention group reported a significantly higher QoL score of 14.444 units compared to the control group. Additionally, the care burden score before the intervention and the group variables were effective on the care burden scores after the intervention, so the intervention group had a significantly lower score of 14.222 points than the control group. (Table 5)

**Table 5:** Effect of supportive care program on care burden and QOL after eliminating the effect of pre-test mean scores

Variables			β	SE	t	*p*
Quality of life	Constant value		14.968	3.736	4.007	< 0.001
	Mean score before intervention		0.758	0.050	15.321	< 0.001
	Group	Control	Ref			
		Intervention	14.444	1.118	12.917	< 0.001
Care burden	Constant value		73.172	4.284	10.078	< 0.001
	Mean score before intervention		0.532	0.048	11.098	< 0.001
	Group	Control	Ref			
		Intervention	−14.222	1.179	−12.063	< 0.001

P: P-value; SE: standard error

## Discussion

The present study investigated the impact of a care program based on a smartphone application on the care burden and QoL of the family caregivers of patients with MDD. The main findings of the present study showed that providing support based on smartphone application has a significant effect on enhancing the quality of life and alleviating the care burden among caregivers of patients with MDD.

The importance of increasing awareness among caregivers by providing access to reliable resources has been highlighted in the literature [46–48]. The care burden can be reduced by focusing on the needs of patients who receive major care at home from their families as well as the needs of their families. Caregivers’ educational needs play a key role in patient recovery. Therefore, increased attention to methods that are suitable for training can affect the caregiver’s care burden [49, 50]. Generally, information is constantly updated by using applications. Mobile application users usually find this method to be effective, efficient, and easy to use. In addition, the use of smartphone applications saves caregivers time. The educational content of the application is also constantly available to caregivers, and the use of multimedia improves the users’ understanding [51, 52].

In the current study, by using the available resources, a reminder, as well as a communication section with nurses, was created for application support. Overall, the use of a reminder in an application can result in greater caregiver satisfaction and reduce the caregiver’s care burden [53]. However, in another study, which provided psychological skill training for caregivers using a web-based application (supported by a nurse phone call), the care burden of cardiac patients did not diminish significantly [54]; this finding might be attributed to the web-based design of the application and the lack of an accessible version. Generally, access to content is one of the important factors in continuing education, as individuals need to review the information. On the other hand, the cost-effectiveness of application-based interventions is one of the reasons for the observed difference between the two intervention methods (web-based and application-based) because the caregiver is required to be online at certain hours of the day to participate in web-based classes (as part of the web-based program), which can influence the care burden in terms of time management. Nevertheless, limited studies have focused on the caregivers of patients, especially the caregivers of patients with mental disorders, using mobile application methods.

By emphasizing the needs of family caregivers, their satisfaction with the environment and physical conditions can be promoted, and the mental and psychological status of patients can be improved, thereby increasing their QoL. In many cases, the caregiver can provide patient care; however, in some cases, they need expert guidance on health issues. Therefore, addressing these needs can successfully increase their understanding of the situation and enhance their coping skills [23, 55]. In the present study, remote caregiver support was provided by a smartphone application. In the design of this tool, in addition to considering the caregivers’ needs concerning patient management, the caregivers’ QoL was addressed. In a similar study, using a web-based application, patient care-related skills were trained using text, videos, interactive exercises, and suggested visits to other websites. However, unlike the current study, this intervention could not improve the caregivers’ QoL [56].

In another study, an intervention using a web-based application could improve the caregivers’ QoL; the efficacy of this intervention was attributed to its high flexibility in matching the caregivers’ plans, in addition to its visual appeal. Although there are many similarities between web-based and smartphone-based programs, the observed differences may be related to their content structure and applications. Moreover, the interaction between the nurse and the caregiver, which is one of the important factors in supporting the caregiver, should be highlighted on different platforms. Overall, the QoL of caregivers can be improved by reducing the care burden. Since an inverse mutual relationship has been proposed between the care burden and QoL in several studies [57, 58], nursing support can be an important source of support for caregivers, increasing their satisfaction and QoL [59].

The results of the present study can help review psychiatric nursing care in patients with major depressive disorder with a supportive approach using a smartphone application. Therefore, it is recommended to use smartphone applications in addition to conducting more studies in the future. The main strength of this study was the authentic educational content of the application, which met the needs of the caregivers and patients, along with the use of smartphone application potentials to support the caregivers. Communication between the nurse and the caregiver, even when they are not present in the treatment setting, may be one of the effective factors. However, this study had some limitations. The knowledge of caregivers toward necessary information in the context of depressive disorders and their related caregiving was not assessed in the current study, so it is recommended to assess this variable in future studies. In general, the main limitation of this study is the lack of a long-term follow-up period of at least three months after the end of the intervention. Regarding dependent variables needs a longer time to change. The next main limitation is comparing the effect of training with smartphone applications in the intervention group with the control group that did not receive the minimum training in Iran. To determine the impact of the application, the training through the application should be compared with one of the traditional training methods such as face-to-face. Since the present study was carried out in one of the main psychiatric referral centers in the Iranian community, the generalizability of the results was limited. Despite the implementation of the intervention in caregivers, patient-related aspects were not measured. Since the study sample had basic media literacy, no new obstacle was encountered during the study; however, in studies on larger populations, some people may not be able to use the application.

## Conclusions

Considering the positive and significant effect of the application on the care burden and the quality of life of family caregivers of patients with major depression, as well as the convenience and applicability of this program, its use is beneficial for major depression patients. This program can have favorable effects on obtaining favorable health outcomes, increasing the ability of the caregiver, and improving the relationship between the caregiver and the nurse. Paying attention to the family is a very important source of support for mental illness, but very few studies have been conducted on this group of people in society to solve their problems and issues.

## Data Availability

All data generated or analysed during this study are included in this published article.

## Abbreviations

MDD: Major Depressive Disorder
QoL: Quality of life
CBI: Caregiver Burden Inventory
WHOQOL-BREF: World Health Organization Quality of Life Questionnaire-Short Form
HTML: HyperText Markup Language
CSS: Cascading Style Sheets
Ajax: Asynchronous JavaScript and XML
PHP: Hypertext Preprocessor
MySQL: My Structured Query Language
MARS: Mobile App Rating Scale
COVID-19: Coronavirus Disease of 2019
ANCOVA: Analysis of Covariance
SPSS: Statistical Package for the Social Sciences
